# Complete chloroplast genome sequence and phylogenetic analysis of wasabi (*Eutrema japonicum*) and its relatives

**DOI:** 10.1038/s41598-019-49667-z

**Published:** 2019-10-07

**Authors:** Natsuko Haga, Masaaki Kobayashi, Nana Michiki, Tomoyuki Takano, Fujio Baba, Keiko Kobayashi, Hajime Ohyanagi, Jun Ohgane, Kentaro Yano, Kyoko Yamane

**Affiliations:** 1 Gifu University, Faculty of Applied Biological Sciences, 1-1 Yanagido, Gifu City, Gifu, 501-1193 Japan; 2 School of Agriculture, Meiji University, 1-1-1 Higashi-Mita, Tama-ku, Kawasaki, Kanagawa 214-8571 Japan; 3 Izu Agricultural Research Center, Shizuoka Prefectural Research Institute of Agriculture and Forestry, 3012 Inatori, Higashiizu-cho, Kamo, Shizuoka, 413-0411 Japan; 4 King Abdullah University of Science and Technology (KAUST), Computational Bioscience Research Center (CBRC), Thuwal, 23955-6900 Saudi Arabia

**Keywords:** Genome, Plant evolution

## Abstract

In Japan, two *Eutrema* species, wasabi (*Eutrema japonicum*, the important traditional Japanese condiment) and yuriwasabi (*E. tenue*), have been recognized as endemic species. We sequenced complete chloroplast (cp) genomes of seven wasabi and yuriwasabi accessions from Japan to study their phylogeny and evolution, using molecular dating of species divergence. Phylogenetic analyses of the complete cp DNA of these two Japanese species and five other Eurasian *Eutrema* species revealed that wasabi and yuriwasabi did not form a monophyletic group. One yuriwasabi accession (Gifu) formed a clade with *E. yunnanense* from China, indicating that this accession should be considered as a different species from the other yuriwasabi accessions. We reveal that Japanese *Eutrema* species diverged from the ‘*E. yunnanense*–yuriwasabi (Gifu)’ clade approximately 1.3 million years ago (Mya), suggesting that the connection between Japan and the Eurasian continent has existed more recently than the Quaternary period. The abundance of cp sequence data in this study also allowed the detection of genetic differentiation among wasabi cultivars. The two polymorphic sites detected between ‘Fujidaruma’ and ‘Shimane No.3’ were used to develop genotyping markers. The cp genome information provided here will thus inform the evolutionary histories of Japanese *Eutrema* species and help in genotyping wasabi cultivars.

## Introduction

Wasabi (Japanese horseradish: *Eutrema japonicum* (Miq.) Koidz., syn. *Wasabia japonica* (Miq.) Matsum.) is a perennial herb that plays an important role in Japanese cuisine and culture. Wasabi paste, made by grating the rhizome, accentuates the taste and flavour of traditional Japanese cuisine such as sushi and buckwheat noodles. The global boom in Japanese cuisine has led to an increase in the consumption of wasabi in many countries. The cultivation of wasabi began approximately four hundred years ago in Japan^1^. The rhizome and the paste products are internationally exported. Around twenty modern cultivars are commonly used. During the history of wasabi cultivation, farmers themselves have improved the agricultural traits of wasabi, thus lineage archives for the modern cultivars rarely exist. Based on interviews with farmers and literature retrievals, Yamane *et al*.^2^ suggests that three major cultivars, ‘Fujidaruma’, ‘Shimane No. 3’ and ‘Mazuma’, have played an important role as mother plants in the development of the modern cultivars. Additional genetic evidence is essential to validate the hypothesis proposed by Yamane *et al*.^2^.

Comparative genome analysis of *Eutrema* species can provide novel insights into the speciation process and the origin of wasabi cultivars. The genus *Eutrema* is mainly distributed in East Asia and has been inferred to be comprised of approximately 26 species^3^. *Eutrema* has attracted increasing attention in recent years, for example, *E. salsugineum* (Pall.) Al-Shehbaz & Warwick is a model plant for the study of salt-tolerance^4^. Among the species in the genus *Eutrema*, *E. japonicum* is recognized as the only cultivated plant. Another Japanese *Eutrema* species, *E. tenue* (Miq.) Makino, is called ‘yuriwasabi’ in Japanese. ‘Yuri’ in Japanese means lily, and the name ‘yuriwasabi’ is derived from ‘lily bulb’. The two species, *E. japonicum* (wild and cultivated) and *E. tenue* (wild only), grow in similar habitats, namely the main island, Shikoku, and Kyushu, and are believed to be endemic to Japan^1^. The species *E. yunnanense*, endemic to China, shares many morphological features with *E. japonicum*. Recent comprehensive molecular phylogenetic studies performed on 183 individuals from 32 species of *Eutrema* using five DNA markers revealed that the closest species to the two Japanese *Eutrema* species is *E. yunnanense*^4^. *E. tenue* (yuriwasabi) is also distributed in China^5^ and was recognized by Yamane *et al*.^1^ based on a plant specimen survey in Kunming and the Beijing Institute of Botany Chinese Academy of Science in 2006. Yamane *et al*.^1^ also noticed that *E*. *yunnanense* is morphologically very similar to *E*. *japonicum*. Therefore, it is important to confirm the level of genetic differentiation and the divergence time between the two Japanese *Eutrema* species and other *Eutrema* species.

Phylogenetic relationships can be studied using genome analyses, and chloroplast (cp) genome sequence analysis is an effective and useful tool in understanding the evolution and phylogeny of plant species. The previous study^1^ provided preliminary evidence that two Japanese *Eutrema* species, *E. japonicum* and *E. tenue*, are native to Japan. These two species formed a monophyletic group in a phylogenetic tree. The analysis employed a partial cp region (ca. 2 kb) for three *Eutrema* species (*E. japonicum*, *E. tenue* and *E. yunnanense*) and several major Brassicaceae species. Further analysis of the speciation and distribution of Japanese *Eutrema* species requires the comparison of many more sequences with intraspecific variations. The complete cp genomes of five *Eutrema* species, *E. yunnanense* Franch., *E. heterophyllum* (W.W.Sm.) H.Hara, *E. botschantzevii* (D.A.German) Al-Shehbaz & Warwick, *E. halophilum* (C.A.Mey.) Al-Shehbaz & Warwick and *E. salsugineum* (Pall.) Al-Shehbaz & Warwick, have recently been reported^6,7^. Comparing these genomes with the complete cp genome data of Japanese *Eutrema* species would greatly advance our understanding of the origin and the speciation process of wasabi cultivars. In this study, we aimed to reveal the intra- and interspecific relationships of Japanese *Eutrema* species, as well as addressing the evolutionary history of Japanese *Eutrema* species using molecular dating of species divergence. In addition, we aimed to develop DNA markers to discriminate among wasabi cultivars using complete cp genome data.

To obtain a comprehensive understanding of the evolutionary relationships and speciation process, we used the Illumina sequencing system to obtain the whole cp genome sequence of the wasabi cultivar, ‘Fujidaruma’, and the Sanger sequencing method to obtain the whole cp genome sequences of four Japanese accessions of *E. japonicum* and two Japanese accessions of *E. tenue*. These accessions cover the geographic distribution of the species in Japan. The DNA sequences in the cp genome of Japanese *Eutrema* were compared with sequences from five other Eurasian *Eutrema* species and the divergence time among the *Eutrema* accessions (defined as cultivars in cultivated wasabi or local populations in wasabi and yuriwasabi) was calculated. In addition, using the comprehensive cp genome sequences, we successfully developed DNA markers to discriminate among cultivars. Increased knowledge of *Eutrema* genetic and phenotypic diversity will not only help to accelerate the breeding of wasabi but will also provide a better understanding of its complex evolutionary process. This is particularly important in times of global warming to which many plant species are responding by shifting their distribution to different latitudes.

## Results

### Characteristics of the chloroplast genomes of *Eutrema* species in Japan

**Figure 1:**
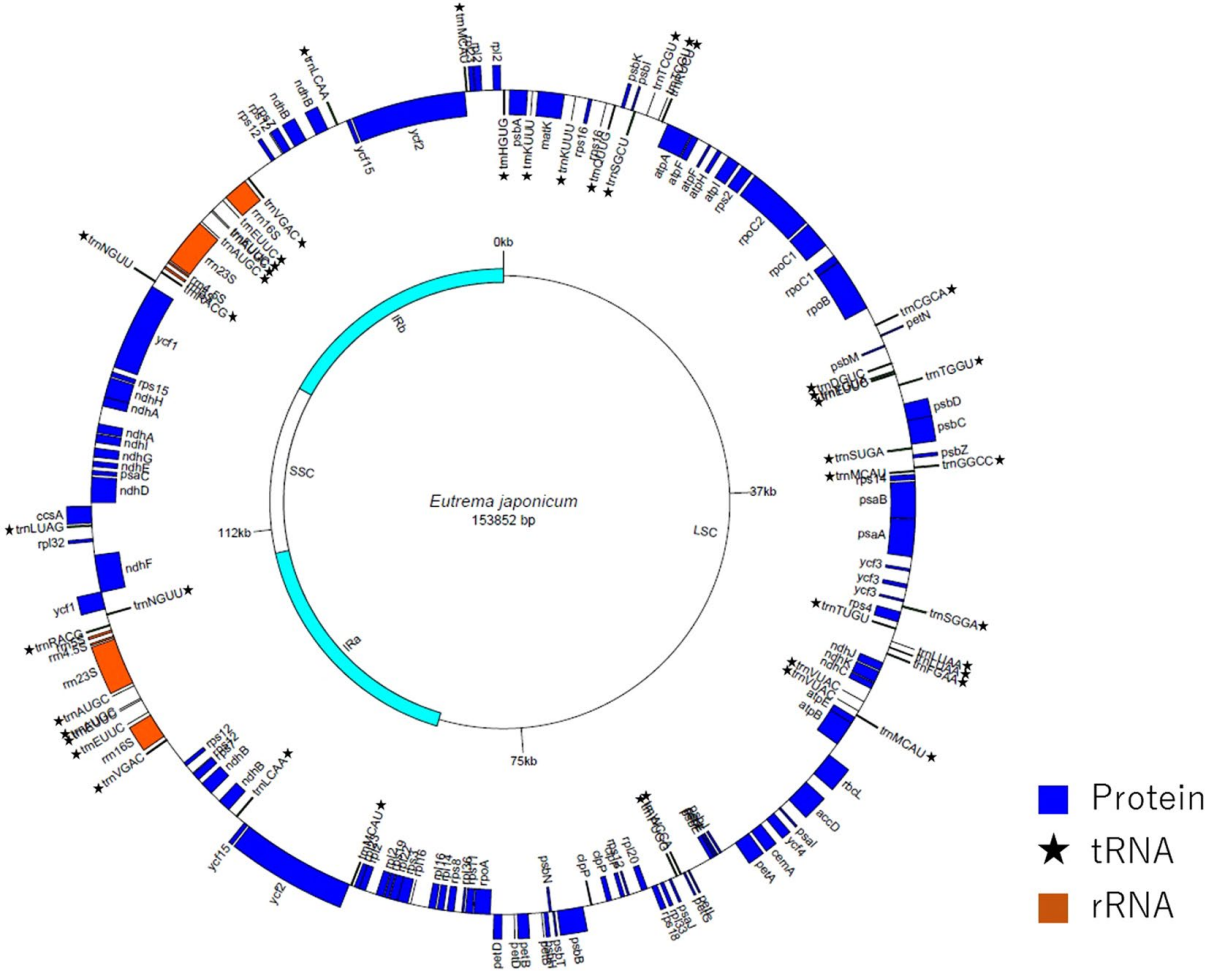
Gene map of *Eutrema japonicum* ‘Fujidaruma’ chloroplast genome. The genes lying outside the outer circle are transcribed in a counter-clockwise direction, whereas genes inside are transcribed in a clockwise direction. Colors and star marks denote the different functional gene groups. LSC: large single copy, SSC: small-single-copy, IR: inverted repeat.

The whole cp genomes of the *Eutrema* species found in Japan were 153,604–153,875 bp long, including the long single copy section (LSC) of 83,879–84,046 bp, the short single copy section (SSC) of 17,715–17,856 bp and a pair of inverted repeats (IRs) of 25,972–26,034 bp each (Supplemental Table S1 and Fig. 1). The cp genomes consisted of 79 protein-coding genes (PCGs) (eight duplicated in the IR), four rRNA genes (four duplicated in the IR), and 30 tRNA genes (seven duplicated in IR). The length of the LSC, SSC, IRa and IRb regions are listed in Supplemental Table S1. The cp gene content of the Japanese *Eutrema* species studied here was completely conserved with other known *Eutrema* cp genomes^6,7^. Summary statistics of the complete cp DNA sequences of the Japanese *Eutrema* species are listed in Table 1.

**Table 1:** Summary statistics of comparative whole chloroplast DNA sequences for Japanese.

		*Eutrema* species in Japan	*E. japonicum*	Three major cultivars	*E. tenue*
		all	all		all
*Eutrema* species					
	Number of samples	7	5	3	2
	chloroplast genome regions				
π (10^−3^)	LSC				
	PCG ^*1^	0.85	0.49	0.15	1.71
	noncoding	2.06	1.28	0.48	3.89
	whole	1.40	0.85	0.31	2.69
	IRa				
	PCG	0.26	0.14	0	0.60
	noncoding	0.25	0.09	0	0.65
	whole	0.23	0.11	0	0.58
	SSC				
	PCG	1.41	0.75	0.35	3.01
	noncoding	3.25	1.63	0.15	7.05
	whole	1.86	0.96	0.30	4.03
	IRb				
	PCG	0.26	0.14	0	0.60
	noncoding	0.24	0.08	0	0.64
	whole	0.23	0.11	0	0.58
	Whole				
	PCG	0.77	0.43	0.14	1.60
	noncoding	1.59	0.94	0.31	3.12
	whole	1.06	0.61	0.20	2.13
Number of Inversion site		11	5	2	8

^*1^protein-coding genes.

### Mononucleotide sequence repeat loci

The minimum number of mononucleotide repeats (MNRs) in the cp genome is two^8^. In the ‘Fujidaruma’ cp genome, about 21,709 A/T and 10,045 G/C sites in the MNR region (repeat number ≥2) were detected in the whole genome. In the LSC region, 12,416 A/T and 4,935 G/C MNRs were identified; in the IRs, 3,188 and 3,192 A/T and 2,129 G/C MNRs were identified in IRa and IRb, respectively. Twenty-nine MNRs (repeat number ≥9) were in 11 genes: *matK*, *rpoC2*, *rpoB*, *atpB*, *accD*, *rpoA*, *rps19*, *ycf2*, *ycf1*, *ccsA* and *rps15*. Detailed MNR location information is listed in Table 2.

**Table 2:** Mononucleotide features of chloroplast genomes in Japanese *Eutrema* species.

		LSC	IRa	SSC	IRb
Region length (bp)		84,006	25,982	17,856	26,008
Number of mononucleotide SSRs (repeat number ≧9^*^)					
	coding regions	12	2	13	2
	intergenic regions	73	5	10	6
	total	85	7	23	8
appearance frequency (10^−3^)					
	for coding regions	0.143	0.077	0.728	0.077
	for intergenic regions	0.869	0.192	0.560	0.231
	for region length	1.011	0.269	1.288	0.308

*The observed mutation rates increase significantly (homopolymer) when mononucleotide.

repeat numbers reach or exceed 9 bp^16^.

### Utility of chloroplast genome sequences at the intraspecific level in wasabi

The previous partial cp analysis showed that the *matK/trnK* sequences (ca. 2 kb) were identical, except for one nucleotide position (a singleton at one accession in *E. tenue*) among Japanese *Eutrema* accessions (‘Fujidaruma’, ‘Mazuma’, and two *E. tenue* accessions^1^). The complete cp genome sequences analyzed here showed a high level of intraspecific sequence diversity within Japanese *Eutrema* species (Table 1). Our data also showed that there were only two polymorphic sites between ‘Shimane No. 3’ and ‘Fujidaruma’. This was not obvious until the complete cp genome sequences were determined. One of these two polymorphic site between ‘Shimane No. 3’ and ‘Fujidaruma’ was 9 or 10 T/A MNR in the intergenic *rpl32- trnL*(UAG) region. Consequently, we successfully genotyped the three major Japanese cultivars. Using this information, we tried to identify the mother line of the unknown wasabi accession, ‘EJ_2016_Kochi’, which has been cultivated without management for at least the last 70 years [personal communication by last author]. We found that the cp genome sequences, excluding the IRa and IRb regions, of ‘EJ_2016_Kochi’ were completely identical to those of ‘Fujidaruma’.

### Phylogenetic analysis of *Eutrema* species

**Figure 2:**
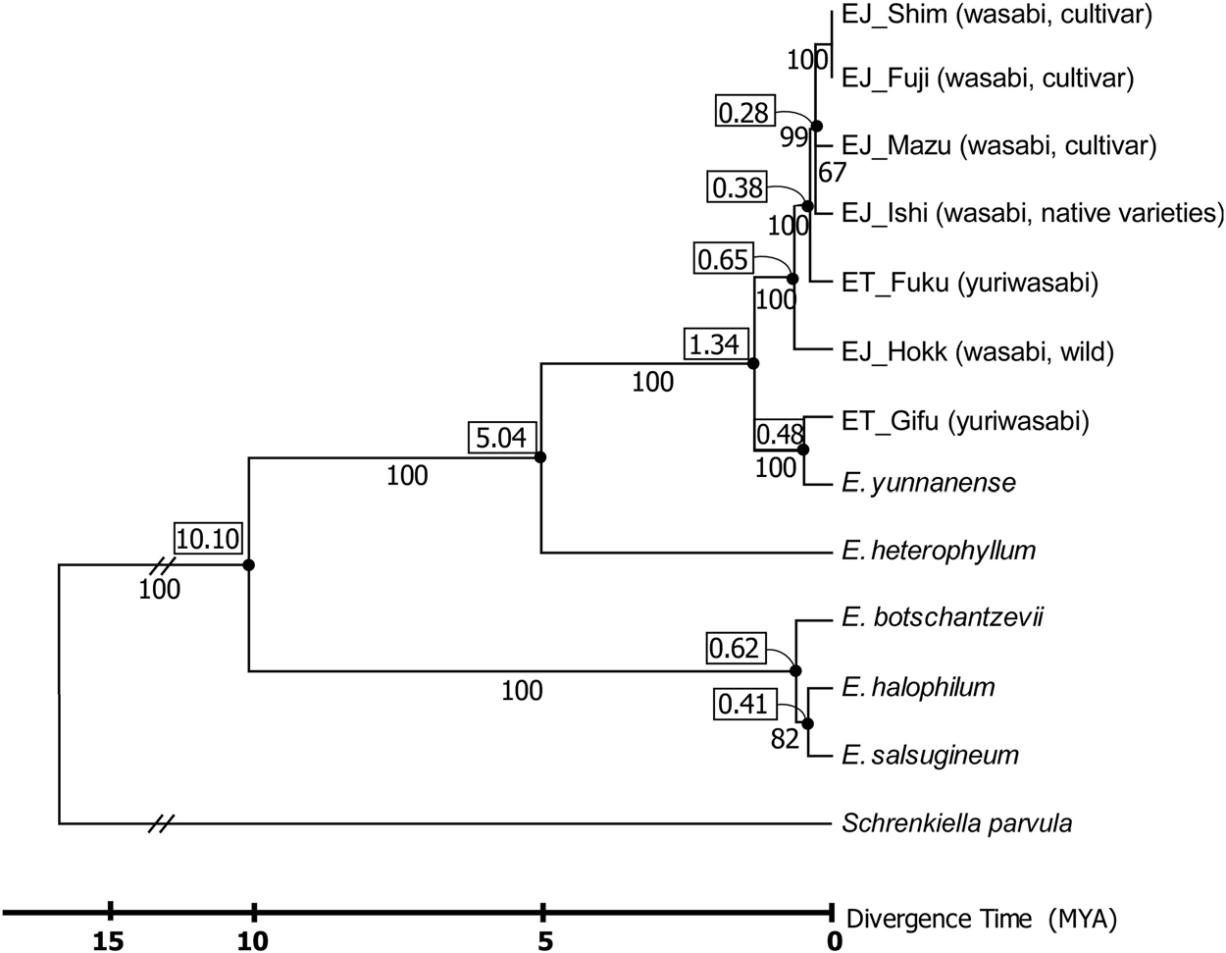
Phylogenetic tree based on 7 *Eutrema* and one outgroup species. The tree is based on 79 chloroplast protein coding genes (PCGs) using maximum likelihood (ML). The ML tree has a ML value of – lnL = 123620.58. Numbers above nodes are bootstrap support values ≥50%. Species and accession names are listed in Table 3. The numbers in boxes represent Mya, millions of years ago.

The data matrix included 79 PCGs from the cp genomes of 13 taxa: five *E. japonicum* accessions, including cultivars, two *E. tenue* accessions from Japan, five other Eurasian *Eutrema* species and one outgroup (*Schrenkiella parvula*). A phylogenetic tree constructed using maximum likelihood (ML) (Fig. 2) showed that: (i) wasabi and yuriwasabi did not form a monophyletic group, (ii) yuriwasabi sampled from Gifu Prefecture grouped with *E. yunnanense* from China (bootstrap values = 100%), (iii) one wild wasabi sample from Hokkaido did not form a monophyletic group with the cultivated wasabi accessions, (iv) the other five accessions of two *Eutrema* species, excluding yuriwasabi of Gifu Prefecture, formed a monophyletic group, and (v), the three major cultivars, ‘Fujidaruma’, ‘Shimane No. 3’ and ‘Mazuma’, were positioned at the most terminal phylogenetic position.

### Divergence time

Based on previous cp *matK* sequences, it is estimated that 4.7 million years (using Nei’s equation) or 5.9 million years (using Bayesian inference) have elapsed since Japanese *Eutrema* diverged from *E. yunnanense*^1^. In this study, the divergence period was estimated using two methods: RelTime-CC and Bayesian inference. Using the RelTime-CC method, the time of divergence between the ‘*E. yunnanense* - yuriwasabi (Gifu)’ clade and the other Japanese *Eutrema* clade was estimated to be 1.34 Mya. Using the Bayesian inference method, the time of divergence was estimated to be 1.5 Mya. The two results are similar, suggesting the data and its interpretation are credible. We also estimated that the Japanese *Eutrema* clade, including EJ_Shim, EJ_Fuji, EJ_ Mazu, EJ_ Ishi and ET_Fuku, excluding yuriwasabi (Gifu) and wasabi (Hokkaido), diverged from the clade consisting of the remaining *Eutrema* accessions approximately 0.65 Mya (Fig. 2). The divergence time for yuriwasabi (Fukuoka) from the remaining four wasabi accessions was estimated to be 0.38 Mya. The terminal branches, including wasabi cultivars, imply that rapid divergence events occurred within approximately 0.4 Mya, in the Quaternary ice age.

### Genotyping

**Figure 3:**
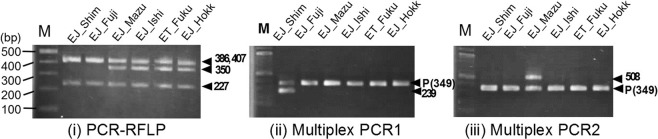
PCR genotyping of the three major Japanese *Eutrema* cultivars. Agarose gel electrophoresis of (i) PCR-RFLP for ‘Fujidaruma’ and ‘Shimane No. 3’, (ii) Multiplex PCR1 for ‘Shimane No.3’, and (iii) Multiplex PCR2 for ‘Mazuma’. Black arrowheads indicate bands of expected sizes. M: Size marker, P(349): Positive *rbcL* (ribulose bisphosphate carboxylase large chain) control amplicon. See Table 3 for the abbreviation of each accession name.

As mentioned above, we successfully genotyped the three major Japanese cultivars, ‘Fujidaruma’, ‘Shimane No. 3’ and ‘Mazuma’, based on complete cp genome sequences. We developed three genotyping markers using cp DNA sequences (Fig. 3): (i) a PCR-RFLP method designed to discriminate between ‘Fujidaruma’/‘Shimane No. 3’ and other accessions. PCR-RFLP produced accurate molecular profiling for ‘Fujidaruma’/‘Shimane No. 3’. The expected band size (350 bp) was found in the other accession samples. Two accessions ‘Fujidaruma’/‘Shimane No. 3’ showed a 350 bp null band (EJ_Shim and EJ_Fuji in Fig. 3); (ii) the genotyping markers were also used with a multiplex PCR to discriminate between ‘Shimane No. 3’ and other accessions. Accurate molecular profiling for ‘Shimane No. 3’ was observed: the expected band size (239 bp) was only found in ‘Shimane No. 3’ (EJ_Shim in Fig. 3); and (iii) a multiplex PCR was also designed to discriminate between ‘Mazuma’ and other accessions. Molecular profiling for ‘Mazuma’ produced the expected band size (508 bp), which was only found in ‘Mazuma’ (EJ_Mazu in Fig. 3). A positive control based on the *rbcL* (ribulose bisphosphate carboxylase large chain) gene was amplified in all the multiplex PCRs, producing an approximately 349 bp band.

## Discussion

Hao *et al*.^4^ reported on the comprehensive phylogenetic relationships of 183 individual plants from 32 species of *Eutrema*, including *E. yunnanense*, *E. japonicum* and *E. tenue*, based on four cp genes: *matK*, *rbcL*, *trnH-psbA*, *trnL-F* and nuclear internal transcribed spacer (ITS) sequences. The results showed a close relationship among these three species and revealed polyphyly of *E. tenue*. Our results showed that yuriwasabi is a non-monophyletic group: one yuriwasabi (Gifu) accession grouped with *E. yunnanense*, supporting the results of Hao *et al*.^4^. However, if we accept that all yuriwasabi accessions, including ET_2014_Gifu, are identical species, yuriwasabi becomes a ‘paraphyletic’ species. If we accept the paraphyletic grouping of yuriwasabi, however, we must reclassify yuriwasabi *E. tenue* as *E. yunnanense*. In order to discuss the reclassification of yuriwasabi (ET_2014_Gifu), we have some evidence to suggest possible reproductive isolation between ET_2014_Gifu and other yuriwasabi accessions. A subset of yuriwasabi populations naturally grows in Gifu Prefecture. For example, there is a yuriwasabi population growing in another valley of the same mountain range as the ET_2014_Gifu population. This population of yuriwasabi represent common morphological features, but ET_2014_Gifu shows some different morphological features, for example, leaves with deeply indented margins and sepal color. Therefore, we propose that the one accession of yuriwasabi which grows naturally in Gifu Prefecture (ET_2014_Gifu) should be considered as a putatively different species. Of course, further taxonomical studies, for example, cross-fertility, morphological and ecological differentiation, are necessary before this proposal can be accepted. Incidentally, the wild wasabi accession from Hokkaido (EJ_2014_Hokkaido) did not form a monophyletic group with other wasabi cultivars (Fig. 2). It is recognized that *Eutrema japonicum* var. *sachalinense* (Miyabe & T. Miyake) Nemoto, known as ‘Karafuto-wasabi’, grows in Karafuto (southern Sakhalin), north of Hokkaido^9^. The present wild wasabi accession from Hokkaido (EJ_Hokk) may need to be reclassified as ‘Karafuto-wasabi’. As we have not yet identified this wild wasabi, further taxonomic studies are required.

Molecular clock analyses of cp DNA data for yuriwasabi (Gifu) suggested a relatively recent divergence between the continental and island species of *Eutrema*: it appears that *E. yunnanense* diverged 0.48 Mya (Fig. 2), which is consistent with the timing of the land bridge formation around the Miocene/Pliocene boundary, at least three times, 1.2 Mya, 0.63 Mya and 0.43 Mya^10^. The opening of the strait may account for climate changes leading to sea level fluctuations during the Quaternary period. The Eurasian continent was connected to Japan by a land bridge when sea levels were 70–125 m lower than present levels, during the last glacial period^11–13^. Currently, the connection between Japanese yuriwasabi (Gifu) and *E. yunnanense* (Chinese) populations is limited by the body of water in the Tsushima strait; however, our result suggests that the connection with the Eurasian continent has existed more recently than the Quaternary period^14^.

The present data demonstrated that the ‘*E. yunnanense* - yuriwasabi (Gifu)’ clade diverged from the other Japanese *Eutrema* clade 1.3 Mya (Fig. 2). A previous study using cp *matK* sequences showed the divergence time as 4.7 million years (using Nei’s equation) or 5.9 million years (using Bayesian inference) (Yamane *et al*., 2016). This discrepancy is probably due to the previous single *matK* gene analysis overestimating the divergence time. Divergence dates estimated from single gene phylogenetic trees can lead to overestimates of divergence times because gene divergence inevitably predates speciation^15,16^. Our present genome sequence data using many genes has provided more informative data. Our phylogenetic data also demonstrated that *E. japonicum* wasabi accessions, including cultivated wasabi, were derived from yuriwasabi (Fukuoka), and speciated from Japanese yuriwasabi 0.38 Mya during the ice age cycles of the Quaternary period (Fig. 2). During this period, endemic Japanese plant species originated, in a group named ‘Sea of Japan Side Element Plants’, and included beech (*Fagus crenata*), *Camellia rusticana* and *Cephalotaxus harringtonia*^17^. The geographical distribution pattern of wasabi is similar to the ‘Sea of Japan Side Element Plants’, in addition, wasabi often grows in beech forests, therefore we suggest that wasabi also belongs to this ‘Sea of Japan Side’ plant group. These species may have rapidly adapted to the Sea of Japan side of Japan and then speciated. It is assumed that the population movement of these species, including wasabi, adapted to this habitat during the Quaternary period. There is no doubt that the Quaternary period was an important age for speciation and diversification in Japanese *Eutrema* species.

Our phylogenetic study also provided important insights on wasabi as a genetic resource. Yuriwasabi has different traits to wasabi, for example, in plant size, flower type, seed size, rhizome shape and life cycle. Our study demonstrated that wasabi originated from yuriwasabi relatively recently, 0.38 Mya (Fig. 2), during the past ice age. Wasabi is mainly located on the Sea of Japan side of Japan, whereas yuriwasabi is mainly located on the Pacific Ocean side of Japan and is distributed at lower altitudes than wasabi. However, natural hybridization between the two species is occasionally found in sympatric habitats, allowing cp DNA introgression. Cultivated wasabi is occasionally transplanted into the natural populations of wild wasabi and yuriwasabi by present-day growers, increasing the chance of hybridization. This suggests that the wild relatives of wasabi are increasingly threatened by genetic erosion. Furthermore, there is evidence, especially in the ‘Sea of Japan Side Element Plants’, that many plant species are commonly affected by global warming. It is predicted that beech (*Fagus crenata*) forests will disappear by 2100 in Kyushu, Shikoku and the Pacific Ocean side of Honshu, and greatly decrease in the Tohoku area of northern Honshu, with extensive loss of the most suitable habitats^18^. The geographical distribution patterns of wasabi are very similar to those of *Fagus crenata* and Japanese blue beech, *Fagus japonica*. Therefore, it is likely that wasabi and its wild relatives will also be affected by global warming. In this case, the utility of wasabi as a genetic resource is threatened.

The phylogenetic tree of wasabi cultivars based on the report of Yamane^2^ demonstrates that almost all current wasabi cultivars are derived from three major cultivars, ‘Fujidaruma’, ‘Shimane No. 3’ and ‘Mazuma’. In this study, we aimed to discriminate between these three mother lines of modern-day cultivars. Complete cp genome sequencing showed that there are only two polymorphic sites between ‘Shimane No. 3’ and ‘Fujidaruma’. The polymorphism between ‘Shimane No. 3’ and ‘Fujidaruma’ had not been detected until the complete cp genome sequences were determined in our study. Previously, it was believed that the mother line of Fujidaruma was the old cultivar ‘Daruma’, which is considered to be a descendant of the first domesticated wasabi accession at Shizuoka Prefecture, about 400 years ago^2^. On the other hand, ‘Shimane No. 3’ originated from a natural hybrid between the native variety ‘Shimane zairai’ (which was semi-domesticated from wild wasabi from Shimane Prefecture, western Honshu, Japan) and the native variety ‘Hanbara’ (which was semi-domesticated from wild wasabi around Tokyo, Japan) in 1942^19^. ‘The two native old Japanese varieties, Shimane zairai’ and ‘Hanbara’ are no longer found in cultivation. There is no data showing that the mother line of Shimane No. 3 is one of these two native varieties. We tried to identify the mother line of ‘Shimane No.3’ using our cp genome data. Presumably, the phylogenetic relationship between ‘Hanbara’ and ‘Fujidaruma’ would be closer than that between ‘Shimane zairai’ and ‘Fujidaruma’, based on the geographical distribution area, because Shimane Prefecture is a long way from Shizuoka and Tokyo. Our study indicated that the cp genome sequences of ‘Shimane No.3’ are very close to those of ‘Fujidaruma’. Therefore, it is highly likely that the mother line of ‘Shimane No. 3’ is not ‘Shimane zairai’ but ‘Hanbara’. In recent years, the importance of local varieties as genetic resources for wasabi has been reviewed. We expect that the development of nuclear markers will provide information on the exploration of ‘Shimane zairai’ and other local varieties as genetic resources.

We also analyzed one wasabi accession, ‘EJ-Kochi’, which had been cultivated without management. This accession has been conserved for at least 70 years but the name of the cultivar is unknown. Partial cp sequences could not identify the name of this cultivar (data not shown). Therefore, in order to identify the standard pedigree line of the three major cultivars, we sequenced the whole cp genome of ‘EJ-Kochi’, excluding the highly conservative IRa and IRb regions. The cp genome sequences of ‘EJ-Kochi’ were completely consistent with the genome sequences of ‘Fujidaruma’.

We also developed three types of DNA markers that could be used to identify the mother line of modern-day cultivars. For example, these markers could be used to identify the cultivar ‘Mazuma’, a very popular and highly priced cultivar. In addition, native varieties, wild accessions of wasabi or escaped cultivars could be identified. Because native varieties are rapidly disappearing, these markers could provide useful information for surveys.

Noncoding cp regions containing chloroplast simple sequence repeats (cpSSRs) represent powerful tools for the detection of intraspecific polymorphism. Yamane and Kawahara^20^ claim that with complete cp genome sequences, cpSSRs with repeat numbers ≥9 would become easy to identify, allowing rapid identification of large numbers of microsatellite loci at relatively low cost (reviewed by Guichoux *et al*.^21^). This would also accelerate ‘genetic barcoding’ and genotyping of plant species. Table 2 shows that cpSSRs with repeat numbers ≥9 were detected in 73 and 10 loci in the LSC and SSC regions, respectively. The cpSSR loci will provide useful information in genotyping studies for native varieties or wild accessions of wasabi. DNA markers developed based on the complete cp genome sequences could identify wasabi accessions and could also be useful for breeding, barcoding, conservation, and/or cultivar assurance.

## Methods

### Plant materials

Samples of two Japanese *Eutrema* species (*E. japonicum* (Japanese horseradish ‘wasabi’) and *E. tenue* (‘yuriwasabi’) were collected from research centres or their natural habitats (Table 3). Samples included six accession lines of *E. japonicum* and two accessions of *E. tenue*. Three out of the six accession lines of *E. japonicum* were the wasabi cultivars ‘Fujidaruma’, ‘Shimane No. 3’ and ‘Mazuma’. For the purpose of genotyping, we additionally analysed one wasabi accession ‘EJ-Kochi’ which has been cultivated without management (using a ‘laissez faire’ approach). Yamane found this accession in 2013 at a remote village in Kochi Prefecture. According to interviews with locals, it was revealed that this accession had been conserved for at least 70 years but the name of the cultivar was unknown. Therefore, we decided to analyse this accession for genotyping.

**Table 3:** Plant materials of *Eutrema* species used in this study.

Species	Accession No.	Abbreviation	Locality/Cultivar, Country	Source
*Eutrema japonicum* (Miq.) Koidz.	EJ_2013_Fujidaruma	EJ_Fuji	Fujidaruma, Japan	Izu Agricultural Reseach Center, Shizuoka Prefectural Research Institute of Agriculture and Forestry
	EJ_2016_Shimane No.3_line2	EJ_Shim	Shimane No. 3, Japan	The Shimane Agricultural Technology Center
	EJ_2013_Mazuma_line2	EJ_Mazu	Mazuma, Japan	Izu Agricultural Reseach Center, Shizuoka Prefectural Research Institute of Agriculture and Forestry
	EJ_2014_Ishikawa_Zairai	EJ_Ishi	Ishikawa Prefecture, Japan	Native to Japan
	EJ_2014_Hokkaido	EJ_Hokk	Hokkaido Prefecture, Japan	Native to Japan
	EJ_2016_Kochi	EJ_Koch	Kochi Prefecture, Japan	Without cultivation management
*E. tenue* (Miq.) Makino	ET_2014_Gifu	ET_Gifu	Gifu Prefecture, Japan	Native to Japan
	ET_2018_Fukuoka	ET_Fuku	Fukuoka Prefecture, Japan	Native to Japan
*E. yunnanense* Franch.	EY		China	GenBank accession No. KT270357
*E. heterophyllum* (W.W.Sm.) H.Hara	EHe		China	GenBank accession No. KT270358
*E. botschantzevii* (D.A.German) Al-Shehbaz & Warwick	EB		China	GenBank accession No. KT962847
*E. halophilum* (C.A.Mey.) Al-Shehbaz & Warwick	EHa		China	GenBank accession No. KT962846
*E. salsugineum* (Pall.) Al-Shehbaz & Warwick	ES		China	GenBank accession No. KR584659
Outgroup species	—		—	
*Schrenkiella parvula* (Schrenk) D.A.German & Al-Shehbaz	SP		China	GenBank accession No. KT222186

### DNA extraction

For sequencing of the cp genomes, young leaves were collected from an arbitrarily chosen individual plant from eight accession lines of *E. japonicum* and *E. tenue*. Total DNA was extracted from the leaves using the method described by Lutz *et al*.^22^.

### Chloroplast genome sequencing of the ‘Fujidaruma’ cultivar

The cultivar ‘Fujidaruma’ was chosen as a representative *Eutrema* accession. To generate high-quality reference genome sequence data, sequence analyses of the cp genome were performed using a HiSeq 2000 (Illumina, San Diego, CA) at Hokkaido System Science Co. Ltd. (Sapporo, Japan) and HiSeq sequencers (Illumina, San Diego, CA) at Meiji University (Kawasaki, Japan).

Approximate 5 μg of DNA was prepared for library construction. Purified DNA from amplified PCR products was fragmented and used to construct libraries according to the manufacturer’s manual (Illumina, San Diego, CA, USA). Extracted DNA was used for the construction of paired-end (PE; insert size of about 300 bp) and mate-pair (MP; insert size of about 3 Kbp) libraries according to standard protocols (Illumina, San Diego, CA, USA).

### Adapter trimming and quality filtering of raw reads of ‘Fujidaruma’ DNA

To correctly perform subsequent *de novo* assembly adapter trimming and quality filtering were performed as described previously^23^. After quality control using FastQC (http://www.bioinformatics.babraham.ac.uk/projects/fastqc/), adaptor sequences were trimmed using Cutadapt (https://cutadapt.readthedocs.io/en/stable/). Low-quality reads were filtered out using an empirically optimized custom Perl script, as follows: (i) both ends of each read had to have QV ≥10 (if not, the end base with QV <10 was trimmed away until QV ≥10 was exposed); (ii) each read must have had an average QV ≥17 (if not, the read was discarded); (iii) the final length of each read had to be ≥20 bp (if not, the read was discarded); (iv) each read had to have had low-quality positions (QV <10) no more than 10% of final length (if not, the read was discarded); and (v) each read must have not contained any N bases (if not, the read was discarded). After adapter trimming, raw reads with lower multiplicity in the k-mer analysis were also removed since the lower multiplicity suggested they were probably caused by sequencing errors.

### Genome assembly of the ‘Fujidaruma’ chloroplast

The assembler tools Platanus^24^ and Ray^25^ were employed and results were compared to obtain reliable genome sequences. Scaffolding of *de novo* assemblies was executed by the tool SSPACE^26^, and GapCloser^27^ was used for error correction. BLAST searches (1e-100) were employed among the scaffolds to select cp genome sequences according to their homology with *E. yunnanense* (GenBan.-KT270357). At present, *E. yunnanense* is the most closely related species to *E. japonicum* (wasabi) among the species whose cp genome sequences are available in the public database.

### Genome annotations of the ‘Fujidaruma’ chloroplast

Repeat sequences were masked by RepeatModeler^28^ and RepeatMasker^29^. Protein-coding sequences in the genome were identified with Maker2^30^ by compiling and analysing datasets from the gene prediction tools Augustus^31^, SNAP^32^ and GeneMark^33^.

### Chloroplast genome sequencing of other plant materials

Nucleotide sequences, excluding ‘Fujidaruma’, were determined using the dideoxy chain-termination method^34^. Based on the ‘Fujidaruma’ sequence of the wasabi cp genome, 12 primer pairs were designed and used for long PCR amplification using PrimeSTAR GXL DNA Polymerase (Takara Bio, Ohtsu, Shiga, Japan) (Supplemental Table S2). A total of 362 sequencing primers were designed and used in this study (Supplemental Table S3). PCR products were directly sequenced using the BigDye Terminator Kit V3.1 (Applied Biosystems, CA, USA). Nucleotide sequences were aligned using MegAlign DNA Star Lasergene 7 (DNASTAR Inc., Madison, Wisconsin, USA) with manual modifications to minimize the number of gaps and minor adjustments. GeSeq^35^ was used to obtain the annotations especially for tRNA (Fig. 1). Nucleotide sequences were deposited in DDBJ/EMBL/GenBank databases under the accession numbers Acc. LC500900 to Acc. LC500908.

### Identification of simple sequence repeats

We investigated the distribution of mononucleotide repeat sequences (MNR) for the purpose of genotyping or DNA barcoding: the most abundant class of simple sequence repeats (SSR) is the mononucleotide (or homopolymer) repeat^36,37^, tracts of identical base pairs (A or T, C or G). A Perl script was used to search for MNR in the cp genome of ‘Fujidaruma’.

### Phylogenetic analysis

Phylogenetic analyses of a 13-taxon data set were conducted based on the 79 chloroplast PCGs, because the alignment of the intergenic regions between *Eutrema* species was difficult owing to the many gaps. The phylogenetic relationships among *Eutrema* species (Table 3) were analysed using ML algorithms with MEGA v. 7^38^ and general time-reversible (GTR)^39^ models of nucleotide substitution. *Schrenkiella parvula* from the DNA DataBank of Japan (GenBan.-KT222186) was used as an outgroup species, based on previous phylogenetic studies^7^.

### Divergence time among *Eutrema* species

In order to check the repeatability of the divergence times obtained, two methods with a relaxed molecular clock were used to estimate the time of lineage divergence: (i) RelTime-CC version 2.0^40^ and (ii) a Bayesian inference framework, implemented using MEGA v. 7^38,41^ and BEAST2 v. 2.4.4^42^. To estimate divergence times using method (i) we used 79 PCGs from cp genomes in *Eutrema*. The ML statistical method based on the GTR-model was used. We used the fossil-based divergence between the ‘*E. salsugineum* - *E. halophylium* - *E. botshantzevii*’clade and the ‘*E. heterophyllum* - *E. yunnanense*’ clade, which was estimated at 10.1 Mya^7^. The BEAST program was used in the Bayesian analyses, creating xml files using BEAUti v. 2.4.4. The rate variation model (relaxed clock:uncorrelated lognormal)^43^ which yielded higher posterior probability estimates was employed. Calculations were based on 1 ×  10^7^ generations, with parameters sampled every 1000 generations using a Yule tree prior. Tracer v. 1.6^44^ was used to evaluate, ensure convergence and effective sample size (ESS) values, density plots and trace plots. Model comparison was conducted by calculation of the Bayes factor based on the relative marginal likelihoods of the models under comparison^45^. Tree files were combined, after removal of 10% burn-in, and a maximum clade credibility tree was constructed using TreeAnnotator v. 2.4.4^42^ to display median ages and 95% highest posterior density intervals (upper and lower) for each node^46^.

### Development of genotyping markers in wasabi cultivars

Three SNP markers were developed from the whole cp genome among the wasabi cultivars for use in this study (Supplemental Table S4). An additional primer set for the ribulose bisphosphate carboxylase large chain gene (*rbcL*) in the cp genome was used as the positive control amplicon, as described^47^.

The specific PCR primer pairs for PCR-RFLP were designed at the coding regions of *ycf4* and *cemA*. The expected length of the PCR products was 1032 bp. The PCR products were digested at two or three sites by the restriction enzyme, *CviKI*-1 (New England Biolabs, Ipswich, MA). As expected from the cp nucleotide sequences, three bands (227, 386, and 407 bp) were generated in ‘Fujidaruma’ and ‘Shimane No. 3’, whereas three bands of 227, 350 and 386 bp in size were detected in the other cultivated accessions (Fig. 3).

Only two polymorphic sites were detected between ‘Shimane No. 3’ and the other accessions, therefore a multiplex PCR marker was constructed within the coding region of the *rpoC2* gene, using specific primer pairs. The expected length of the PCR product was 239 bp for ‘Shimane No. 3’. The 3′-terminal position in the primer is essential in controlling mispairing^48^. A second mismatch was introduced at the third base from the 3ʹ-end of both species-specific primers in order to increase their specificity, because it was reported that a single mismatch primer was inadequate in discriminating between ‘Shimane No. 3’ and other accessions^47,49^. The reverse primer was designed with two mismatch sites for all accessions except for ‘Shimane No. 3’.

Specific PCR primer pairs for the multiplex PCR were designed from the coding region of the *ycf3* gene. The expected length of the PCR products was 508 bp for ‘Mazuma’. The forward primer was designed using an autapomorphic indel specific to ‘Mazuma’.

### PCR amplification and detection

All PCRs were performed as follows: 30 cycles of 45 s at 95 °C for denaturation, 30 s for annealing, and 15 s at 68 °C for polymerization (Taq DNA polymerase; New England Biolabs, Frankfurt am Main, Germany), with a final extension of 3 min at 68 °C. PCR-RFLP and multiplex PCR products were analysed using 1.0 or 1.5% agarose gels stained with ethidium bromide and visualized with UV illumination. Band patterns were confirmed at least twice for each accessioned species.

## Supplementary information

Supplementary_Tables
