# Supplementary material for: Complete chloroplast genome sequence and phylogenetic analysis of wasabi (Eutrema japonicum) and its relatives
Source: Sci Rep. 2019 Oct 7;9:14377. doi: 10.1038/s41598-019-49667-z (PMC6779752; doi:10.1038/s41598-019-49667-z)
Supplement: Supplementary file 1 — Supplementary_Tables [file 41598_2019_49667_MOESM1_ESM.pdf]

Complete chloroplast genome sequence and phylogenetic analysis of wasabi (*Eutrema japonicum*) and its relatives

Natsuko Haga, Masaaki Kobayashi, Nana Michiki, Tomoyuki Takano, Fujio Baba, Keiko Kobayashi, Hajime Ohyanagi, Jun Ohgane, and Kentaro Yano, and Kyoko Yamane*

*Corresponding Author

Gifu University, Faculty of Applied Biological Sciences, 1-1 Yanagido, Gifu City, Gifu, 501-1193, JAPAN

E-MAIL: kyamane@gifu-u.ac.jp

Supplemental TablesS1-S4

Supplementary Table S1. The chloroplast genome of seven *Eutrema* species.

Species	Accession No.	Total size (bp)	LSC (bp)	IRa (bp)	SSC (bp)	IRb (bp)
E. japonicum	EJ_2013_Fujidaruma	153,852	84,006	25,982	17,856	26,008
	EJ_2016_Shimane No.3_line2	153,851	84,006	25,982	17,855	26,008
	EJ_2013_Mazuma_line2	153,794	83,990	25,972	17,834	25,998
	EJ_2014_Ishikawa_Zairai	153,604	83,879	25,992	17,715	26,018
	EJ_2014_Hokkaido	153,844	84,046	25,972	17,828	25,998
	EJ_2016_Kochi	-	84,006	-	17,856	-
E. tenue	ET_2014_Gifu	153,875	84,017	26,014	17,810	26,034
	ET_2018_Fukuoka	153,802	83,998	25,974	17,830	26,000
E. yunnanense	EY	153,948	84,097	26,015	17,801	26,035
E. heterophyllum	EHe	153,876	83,961	26,037	17,815	26,063
E. botschantzevii	EB	153,279	83,816	25,893	17,651	25,919
E. halophilum	EHa	153,311	83,846	25,893	17,653	25,919
E. salsugineum	ES	153,407	83,895	25,917	17,652	25,943

Supplementary Table S2. Long PCR primer sequences.

Primer Name	Primer sequence (5'–3')	*Tm*
LSC_2_F_wasabi_cp_genome	TTTTCTTTCTTCTCTTAGCCTTTGTTTGG	68
LSC_2_R_wasabi_cp_genome	TGCTTTAGAAGAAGCAATCTGTTATCGAG	68
LSC_18_F_wasabi_cp_genome	CTAAATCACTGGTATGACTATCAAAATCGT	65
LSC_18_R_wasabi_cp_genome	TACTAAAGGCACAAAAATAGAGGGTAAGTT	65
IRb_LSC_20_F_wasabi_cp_genome	CTTATTTTACTATGTTCAACTCTCTGTATTCC	62
IRb_LSC_20_R_wasabi_cp_genome	ACTAATTGTTTTACACTTTTTACTTGGTGTAG	63
LSC_22_F_wasabi_cp_genome	TTATAATATGAATACCTTTACGCTTTGCAG	64
LSC_22_R_wasabi_cp_genome	GTTTATTGATCAGGGTTTAATAGAAGAACT	63
LSC_23_F_wasabi_cp_genome	CTTTAAAAGGTGTTGTGAATGAGTTATTTC	64
LSC_23_R_wasabi_cp_genome	ACAAAAGTGTGTAAACTATAAAAACGAGTC	62
LSC_IRa_24_F_wasabi_cp_genome	GAAACCTTGGTTTACCTATCTCAATAAAGT	64
LSC_IRa_24_R_wasabi_cp_genome	AACTATGTGATTGAATAAATCCTCCTCTAT	63
SSC_25_F_wasabi_cp_genome	GAACAAATACAAGAACAGATAAGAAGAGAT	62
SSC_25_R_wasabi_cp_genome	AAACGTGTAGTGGTTTTTACTGATAAAGAG	64
LSC_27_F_wasabi_cp_genome	TTCTGAGGTATATTAAGATTAAGTCTCCAG	62
LSC_27_newR_wasabi_cp_genome	TAACGCTTACTCCTGGTAATTATATTGAAG	64
IRa_SSC_28_F_wasabi_cp_genome	GACACTAATACAATTAGATCTGCTCTTCAT	63
IRa_SSC_28_R_wasabi_cp_genome	ATTTTGGACTTAATACACGAACTTTCTATT	63
IRb_LSC_32_F_wasabi_cp_genome	TGAGATTCATAGTTGCATTACTTATAGCTT	63
IRb_LSC_32_R_wasabi_cp_genome	ACAATTCTCGTTCTTTACATTTCTTCTTAG	64
LSC_33_F_wasabi_cp_genome	ACTAATGCTAAAGAATTATCCATTTGTAGA	62
LSC_33_R_wasabi_cp_genome	CTTTCTCCTTTTGATGATGTGTAATAGATA	63
SSC_IRb_34_F_wasabi_cp_genome	AGTAAGTCTTCGTATCTTATTAGTGAAACTGA	63
SSC_IRb_34_R_wasabi_cp_genome	CTTTGACTTAGGATTAGTCAGTTCTATTTCTT	63

Supplementary Table S3. Sequence primer sequences for sangar methods.

Primer Name	Primer sequence (5'–3')
2_1_wasabi_cp_genome	AACCCTCGGTACGATTAACT
2_2_wasabi_cp_genome	CCGATTACTTTATTCGACAA
2_3_wasabi_cp_genome	CTCCATCCATCATAAAGGAG
2_4_wasabi_cp_genome	TATCATTTCTCCTGAAGTATAA
2_5_wasabi_cp_genome	ACATTTGTCGATAAGCTTGT
2_6_wasabi_cp_genome	AAGACCATAAATACGAGCGA
2_7_wasabi_cp_genome	ATGCTCACTTATTCCAATCA
2_8_wasabi_cp_genome	ATATCGGTATTAAACCCGAA
2_9_wasabi_cp_genome	AGCTGTACCTTGACCAACTC
2_10_wasabi_cp_genome	CCACTAAAGGTACTAAAGAAAC
2_11_wasabi_cp_genome	TTTACTTGAATTTGATATTGTAG
2_12_wasabi_cp_genome	TGACTCTTCTATTTAAAAATCTG
2_13_wasabi_cp_genome	AACATATCTAGGCGGGATTA
2_14_wasabi_cp_genome	AATTGCTATCTTATTAGGTTCC
2_15_wasabi_cp_genome	GTTAAAGATCTATATAATCTGCC
2_16_wasabi_cp_genome	TATTCATTGAGACAACAATCC
2_17_wasabi_cp_genome	TACTTTGATTCTCTATACAACAA
2_18_wasabi_cp_genome	CGTGAATGGATACTCTGAAAT
2_19_wasabi_cp_genome	AGCTTCTGAATCTCGGTTAA
2_20_wasabi_cp_genome	ATTAATGTAGGGATTTCTGTCTC
2_21_wasabi_cp_genome	GAATAGAATATATAGAAATGGAA
2_22_wasabi_cp_genome	TCTTTAGTAAACCAAAGTTCTC
2_23_wasabi_cp_genome	AGGATTCTCTATTTCTATTATTG
18_1_wasabi_cp_genome	TAGAGAATACCAGATGAAATAGA
18_2_wasabi_cp_genome	GTAACTATCCCGCTTTCATA
18_3_wasabi_cp_genome	TTTAACGAATTCTCCGTTTC
18_4_wasabi_cp_genome	CTCTGTAATAAGTAAATGCCTC
18_5_wasabi_cp_genome	GTTTGTCCGAACTCTTGTTT
18_6_wasabi_cp_genome	TGATAATGATAAATGACGTG
18_7_wasabi_cp_genome	CTTATCTGATTTGAAATTCTTG
18_8_wasabi_cp_genome	GTCTGATAGATCCTTAGTGGA
18_9_wasabi_cp_genome	CACATAATACATATTCGAAAGG
18_10_wasabi_cp_genome	TGATACACACTGGCTAAGAG
18_11_wasabi_cp_genome	GCATTTGTTCATATAATCTCAC
18_12_wasabi_cp_genome	CGTAAAGCAGAAACATAGAC
18_13_wasabi_cp_genome	GATAGTGATTGGATCCATTG
18_14_wasabi_cp_genome	GGTACTTTAATAGGACCTAATG
18_15_wasabi_cp_genome	TGTGGATCAATGTCACTATTC
18_16_wasabi_cp_genome	TCTTGATAGTACGGTAGTCG
18_17_wasabi_cp_genome	ACCTAAATTATCAACAGGTTC
18_18_wasabi_cp_genome	CATTATTTCGATCTCATTAGC
18_19_wasabi_cp_genome	GTTACAAAGGACGATGCTAC
18_20_wasabi_cp_genome	CGTACTAGCTAAAGCTTTACG
18_21_wasabi_cp_genome	GTAAAGTTGTGTCCACAAGAAC
18_22_wasabi_cp_genome	GGAATTTCATATCGGATGAC
18_23_wasabi_cp_genome	TGTTCAAACAGGTACAGGTC
18_24_wasabi_cp_genome	GATTACGTGGTACAATTGCA
18_25_wasabi_cp_genome	ATTGAATCCACAATTGATTG
18_26_wasabi_cp_genome	AATAGATGAATAGTCATTCAATG
18_27_wasabi_cp_genome	CTAGTTCAGGACTCCATTTG
18_28_wasabi_cp_genome	ATAAGAAACGGTCTCTCCAAC
18_29_wasabi_cp_genome	AACAACAAGGTCTACTCGAC
18_30_wasabi_cp_genome	TCTGTATTCGAAAAATTTCG
18_31_wasabi_cp_genome	AAGAATCTGGAGTCATTAATG
18_32_wasabi_cp_genome	TCTAGCGGAAACAATTAGAG
18_33_wasabi_cp_genome	TACCTACTATTGGATTTGAACC
18_34_wasabi_cp_genome	CTTTGGTAGTTCTTCTGTATTG
18_35_wasabi_cp_genome	GTAAGATGGCTATGGTAAAGTC
18_36_wasabi_cp_genome	CGTGCTTATCCTAATTCTTG
18_37_wasabi_cp_genome	GGAAATTATGATCAAGAATTAC

18_38_wasabi_cp_genome	AACGAGTCTTAGCTTCTACAAC
18_39_wasabi_cp_genome	AAGAAACGCAATAGTAGTAAATG
18_40_wasabi_cp_genome	TCATTATGATGTCTTTCCAC
18_41_wasabi_cp_genome	TTAGTGAATTTCTATGTATGCG
18_42_wasabi_cp_genome	TACTTAAATATGTTCGTATCGC
18_43_wasabi_cp_genome	AATTAGTTGAAAGTACGGAAAG
18_44_wasabi_cp_genome	TATACGAATAGGAGGCACAG
18_45_wasabi_cp_genome	TCATTACGTGCGACTATCTC
18_46_wasabi_cp_genome	AACAAAAGAGTTGGTTGTCG
20_2_wasabi_cp_genome	GAAGAAGCTTGTACAGTTTG
22_1_wasabi_cp_genome	TAACAGATTGCTTCTTCTAA
22_2_wasabi_cp_genome	CGTTTTCATCGATTAAATAAG
22_3_wasabi_cp_genome	ATACTGTCCGCTATCTCATAG
22_4_wasabi_cp_genome	GCAGAATATATGAAATTATAATG
22_5_wasabi_cp_genome	GCAATAATACCTACAGCTTC
22_6_wasabi_cp_genome	GTACATTCCCATAATGATGC
22_7_wasabi_cp_genome	ATAAGCTCCAATTGCATCATA
22_8_wasabi_cp_genome	AATGACTTGTAAACCTTATTATG
22_9_wasabi_cp_genome	GGCTCAAGTAGTTATACCAA
22_10_wasabi_cp_genome	AATTTCGATCAAATCACACA
22_11_wasabi_cp_genome	AATATGAGCAACACCAAACC
22_12_wasabi_cp_genome	AAGTATAGCCCGTAATAATCTA
22_13_wasabi_cp_genome	AATATTGGAAAGGAAAAATG
22_14_wasabi_cp_genome	TCGTGTGAGTAGGAAACAGA
22_15_wasabi_cp_genome	GAACTGCTAATTTTCGGATAC
22_16_wasabi_cp_genome	GTAAATGCATTAAAAAGGGA
22_17_wasabi_cp_genome	AGATATTTGAATGCTCGCTT
22_18_wasabi_cp_genome	AATACCGAATGGAGAGATAG
22_19_wasabi_cp_genome	AATATTGTGGCATAACCATC
22_20_wasabi_cp_genome	GAAGATCGAAAAATTGTAAGA
22_21_wasabi_cp_genome	GATTAGGCATCCAGTCATTC
22_22_wasabi_cp_genome	ATTCAACTCGGACCATAGTT
22_23_wasabi_cp_genome	AGTTGTAGATACTGCGGTACG
22_24_wasabi_cp_genome	ATTCGTGAGGGAACATACAC
22_25_wasabi_cp_genome	ATACGAAATGAAAGTGGATC
22_26_wasabi_cp_genome	ATTGATCTCTGCAGATCACA
22_27_wasabi_cp_genome	TATATGAAAAATCGAGATCC
23_1_wasabi_cp_genome	TCAGAATATAACAATAACAGGTA
23_2_wasabi_cp_genome	TTCCAGTTATCTTGGTAGAA
23_3_wasabi_cp_genome	TCTGAGTTATTCTTTCTAGATTC
23_4_wasabi_cp_genome	GGGAACTAATGATTGGTTATA
23_5_wasabi_cp_genome	ACAAGTTCTAGCTAATGGTAAA
23_6_wasabi_cp_genome	TTAGCTTTATCAAATTCGTAAA
23_7_2_wasabi_cp_genome	TCTGATGATAAAAAATATTGTGT
23_8_wasabi_cp_genome	AGAAACGGTAGGTACAGCTA
23_9_wasabi_cp_genome	CAAGAGGTTGGTTTCTAATTA
23_10_wasabi_cp_genome	AAGTTCTATGTTTTGACTGAAG
23_11_wasabi_cp_genome	TTTACAATACAGACGTGGTG
23_12_wasabi_cp_genome	GGAATCTATAAGATCGTTCTAG
23_13_wasabi_cp_genome	GGAATGTACCAGTTGTGTTC
23_14_wasabi_cp_genome	GACTTATTCTTCAATTGAATCAC
23_15_wasabi_cp_genome	CCTTTACTAGTCATTAATAACCA
23_16_wasabi_cp_genome	TAAACGAGTCGCACATACAC
23_17_wasabi_cp_genome	CCAAATATAGATTGTATTATACG
23_18_wasabi_cp_genome	ATAAAGTCGGTTGATTAGGATA
23_19_wasabi_cp_genome	TTATTAGTCCGATCACGTAAAC
23_20_wasabi_cp_genome	TTCCAATGCAATAAAGTTAC
23_21_wasabi_cp_genome	CTTATTCCATCTTAGTGTTCG
23_22_wasabi_cp_genome	AGTCCAAGAGGTTGGTTTAC
23_23_wasabi_cp_genome	TATGCTAAGATCCTTTATTTACA
23_24_wasabi_cp_genome	AGGCTTCTTTGTATTTTATG
23_25_wasabi_cp_genome	CTCATTCAATATTATTTAAAG

23_26_wasabi_cp_genome	GAACTACCAAGGGAGAATAG
23_27_wasabi_cp_genome	TGTATAAACCAATGCTTCCA
23_28_wasabi_cp_genome	CTAACCATCCAACAGCTATT
23_29_wasabi_cp_genome	AAGCCAGAAAACACGATATG
23_30_wasabi_cp_genome	AATAAAGTAGGAAAAAAGGATAA
23_31_wasabi_cp_genome	GTAGAGCATAAACCTAAAAATG
23_32_wasabi_cp_genome	TTGAGAAAAACAATACTATGC
23_33_wasabi_cp_genome	GAAAGGGATGTTTTTATGTC
23_34_wasabi_cp_genome	CGTTACTGTATAAAGTGGATC
23_35_wasabi_cp_genome	GCAATTACTTCTTAATCGGA
23_36_wasabi_cp_genome	ATTTTGATCGGATCAAAATC
23_37_wasabi_cp_genome	ATCGTTGATCGTATCTCTAA
23_38_wasabi_cp_genome	GTATATACGTACAAAATCGATTC
23_39_wasabi_cp_genome	CATATATTTCTAAAGTACTTCCC
23_40_wasabi_cp_genome	AACCTTTGAATTGAATACAAC
23_42_wasabi_cp_genome	TATGGTTCATATTCAGGATTA
23_43_wasabi_cp_genome	TTGCTTTCCTTATCTTATTTTAG
23_44_wasabi_cp_genome	ACCATCAGGATAAATCTGAC
23_45_wasabi_cp_genome	TGTTGAAATATAAAAGGAATC
23_46_wasabi_cp_genome	TAGTAATCCAAGTTTTCAAAC
23_47_wasabi_cp_genome	GAATTTCCATATAAAGGACA
23_48_wasabi_cp_genome	CCAGATAATACGTTTACACA
23_50_wasabi_cp_genome	CATTGAGTTCTTATGTTTTCA
24_1_wasabi_cp_genome	TTTCTCTTATTACTTGGTGAA
24_2_wasabi_cp_genome	TCGTTAGTTGCAATAACTTTG
24_3_wasabi_cp_genome	GTATCTAGGGAGTAGTCATTTC
24_4_wasabi_cp_genome	ATTCAATACACAAATCTATTGG
24_5_wasabi_cp_genome	TACTGCTCGAATAGCATTTC
24_6_wasabi_cp_genome	ATATGTCGAATCCTTTCAGAAG
24_7_wasabi_cp_genome	CCTTAATTGTTCTATAATTTTG
24_8_wasabi_cp_genome	TATTCAGAAATAATGAATTGG
24_9_wasabi_cp_genome	AGTTAGAATATTCTAACAAATCC
24_10_wasabi_cp_genome	ACAATGATATGATCGGTCTA
24_11_wasabi_cp_genome	TTGAACATTCATCTGTAATTC
24_12_wasabi_cp_genome	GACATAGATATATATTGCCCTA
24_13_wasabi_cp_genome	CAATGAAAACATAAATCTAACA
24_14_wasabi_cp_genome	TGGATATTCGTAGATGAAGA
24_15_wasabi_cp_genome	TGTAAGAGCTCCATTGTAGAA
24_16_wasabi_cp_genome	GCTTGGATCACATCTAGACA
24_17_wasabi_cp_genome	CATTGATCAAGAAGGAAATC
24_18_wasabi_cp_genome	AGACCGAGAAGCTAGACTAA
24_19_wasabi_cp_genome	AAGTCTGTTTTGACATGAAATG
24_20_wasabi_cp_genome	AGCTTTACTTGGCGAAATAG
24_21_wasabi_cp_genome	CCAGAATCTATAATTGTCTCAA
24_22_wasabi_cp_genome	ATGGTACCAAGAATGACTAC
24_23_wasabi_cp_genome	GTCGGGTATGATCATAGAAA
25_1_wasabi_cp_genome	GTTAATAATGGTACTAACCCAAA
25_2_wasabi_cp_genome	CATGTATAAGAGCCGAAATC
25_3_wasabi_cp_genome	AAGTAGAAGTCCAACTCCTAGTA
25_4_wasabi_cp_genome	GCAAGAAATATTATTATAATCGA
25_6_wasabi_cp_genome	ATCGGGTAAAACCTAGTATTAA
25_7_wasabi_cp_genome	TTATAGGTATCAATTGATTGAAC
25_8_wasabi_cp_genome	TTGACATTAATTAACTTGTATCC
25_9_wasabi_cp_genome	AATAGTATTTCGAGTGTGACAG
25_10_wasabi_cp_genome	TTGAGTACGAGTACTTGTCAA
25_11_wasabi_cp_genome	CCTTTTAGTTGGGAATTATC
25_12_wasabi_cp_genome	ATCTGTGGAATATTCTGAATC
25_13_wasabi_cp_genome	ATTCGTGACGATCATAAGTT
25_14_wasabi_cp_genome	TAGCTCCAGAATAGATATATACG
25_15_wasabi_cp_genome	ATTCATGACAGAAATTCTATTT
25_16_wasabi_cp_genome	GAATCGCACGTAGAGATATT
25_17_wasabi_cp_genome	AACCACTTTGATCTCCTATG

25_18_wasabi_cp_genome	ATGAGAAGCTATACGACTCAA
25_19_wasabi_cp_genome	TGAAATGAAATGAATGCATT
25_20_wasabi_cp_genome	TTATGAACCAATGAAATACCTAT
25_21_wasabi_cp_genome	TACGATGATTTAGTATTTCTTCA
25_22_wasabi_cp_genome	GTTATATTTAAGAACAGCCTTTA
25_23_wasabi_cp_genome	AGCAAGTTTTTGATAATCCA
25_25_wasabi_cp_genome	AATTTCCAAGAAACTGGTTA
25_26_wasabi_cp_genome	ATATCCGATTAATTCGTAAATC
25_27_wasabi_cp_genome	TTGCTACAAATCTATTCATTCTA
25_28_wasabi_cp_genome	TTATTTCTCACAAGGTATTCG
25_29_wasabi_cp_genome	TTAAATTCATTAATGATAGATCC
25_30_wasabi_cp_genome	CAGGAAAAGATCTTATGATAGTC
25_31_wasabi_cp_genome	TATGAGTCTTACGATGAATTTG
25_32_wasabi_cp_genome	GGAATTCTTCAAGCTCTAGC
25_33_wasabi_cp_genome	GTATTATAAAGTGAGCGGCA
25_34_wasabi_cp_genome	AATAGCGGCGAGAATAGTAA
25_35_wasabi_cp_genome	CTACGAGCTGCAAGGTATAT
25_36_wasabi_cp_genome	TCCATTAGATTCTAATTTCACTC
25_38_wasabi_cp_genome	ATTGAAATTTTATGACATTCAA
25_39_wasabi_cp_genome	TTACTGACGGGATTTATTACTAC
25_40_wasabi_cp_genome	TATTGGTTCTATAACGGATC
25_42_wasabi_cp_genome	AGAGCTGGTACTAATATTGCA
25_43_wasabi_cp_genome	CAATGGAATTTACAACTTATTTC
25_44_wasabi_cp_genome	TTGAATTACCTGTAGAAAGTG
25_45_wasabi_cp_genome	CTGTATCTGCATAAAATCACA
25_46_wasabi_cp_genome	ACTTCAATGTTAGGATTAGTTAC
25_47_wasabi_cp_genome	TTCAATGGAAGCTATAGTTG
25_48_wasabi_cp_genome	AGATTGGTATGAATTTTTGAA
25_50_wasabi_cp_genome	GTTATGGTTCATTTACATCAA
25_51_wasabi_cp_genome	GTTTTACCCGATTTGAGAAA
25_52_wasabi_cp_genome	TACATCCTATACAGGTATCATAA
25_53_wasabi_cp_genome	GTATTGTTTTGAACAAGTTG
25_54_wasabi_cp_genome	TGAGTTTGATTATTTAGAATTATT
25_55_wasabi_cp_genome	CGACCTTATTAAATAAGTAGCA
27_1_wasabi_cp_genome	AATTTCGTGATCTATATCTTCTA
27_2_wasabi_cp_genome	TAGGTAAGAAAACTTGTGTGTT
27_3_wasabi_cp_genome	AACGTAGGAAAAGATTCTTG
27_4_wasabi_cp_genome	CTCTATCAACCTGTATCAATTA
27_5_wasabi_cp_genome	CTTGTAAACTCACTCAGATTAA
27_6_wasabi_cp_genome	CGTCCAGTATCTCTTTCTTG
27_7_wasabi_cp_genome	AGGGTTATGTAGACAGCGAA
27_8_wasabi_cp_genome	TCATTGCATGGATATTTATAA
27_9_wasabi_cp_genome	TTCCTGCCTAGAGAAAGAAA
27_10_wasabi_cp_genome	TATGACTATAGCCCTTGGTAA
27_11_wasabi_cp_genome	ACTCAAGCCGAAGAAACTTA
27_12_wasabi_cp_genome	CGAAACTCTTGAAGAATCTT
27_13_wasabi_cp_genome	TAACGGTTTGGACTTGAGTA
27_14_wasabi_cp_genome	TAAATGAGATACACGATCCA
27_16_wasabi_cp_genome	TTACCAGCTTGATCTTGTTG
27_17_wasabi_cp_genome	CCAGACTGATAAACTATTCATAC
27_18_wasabi_cp_genome	TCTAACATTCTTGCCAATAC
27_19_wasabi_cp_genome	ACAAATGATGATTCAGACGA
27_20_wasabi_cp_genome	TATTATGAGCCCAAACAATG
27_21_wasabi_cp_genome	ATCTGAAAACATATCTTGTGG
27_22_wasabi_cp_genome	TCATGAGGAAGTGGTATTTC
27_24_wasabi_cp_genome	CCTATGGATATGTCATAATAGA
27_25_wasabi_cp_genome	ACAACTTTATTGTACCGCAA
27_26_wasabi_cp_genome	CATTGTTCACACATCACATG
27_27_wasabi_cp_genome	GGGTGGTAACTCATATTACT
27_28_wasabi_cp_genome	TTACATGTAAGACCGATTGC
27_29_wasabi_cp_genome	GCATAAGGGTCTTTATGACA
27_30_wasabi_cp_genome	TTCCAAGTTAATGCCAGATA

27_31_wasabi_cp_genome	ACTGAAGTATTATGGCAAAG
27_32_wasabi_cp_genome	TGAATCCATTTATATTTGGG
27_33_wasabi_cp_genome	ATCAGGAGACGCAAATACAA
27_34_wasabi_cp_genome	AACCGATTTTGACTTTCCTA
27_35_wasabi_cp_genome	CCAGATTCCACCAAATATAC
27_36_wasabi_cp_genome	AGAACCTCCTCAGGGAATAT
27_37_wasabi_cp_genome	ACAGACCACCTAATTGACAC
27_38_wasabi_cp_genome	ATCTACATGTACATACCCTTATG
27_39_wasabi_cp_genome	AATTAGAAATAATACACTTTCGA
27_40_wasabi_cp_genome	ATAAGCTAGTCATACCATTTG
27_41_wasabi_cp_genome	GAAGGAATATGGAAGCTATC
27_44_wasabi_cp_genome	CAAAGTATCCAAGGAATCTG
27_46_wasabi_cp_genome	TATAGTCTCCGTTCATACGTA
27_47_wasabi_cp_genome	GGATTTAAGTATCCCTTAATT
27_48_wasabi_cp_genome	GATATGGGTGGATTCCTTTA
27_49_wasabi_cp_genome	CCTTTCGTTTTACTTCCAAA
27_50_wasabi_cp_genome	ATCCAACTCCAAGATATATG
28_1_wasabi_cp_genome	CATATAATCGTGGTTACATAGAT
33_1_wasabi_cp_genome	TAACAAGCTACACCAAGTAA
33_2_wasabi_cp_genome	CTAGTCGGATGGAGTAGATAA
33_3_wasabi_cp_genome	CAACGATCCAATAATAGAAG
33_4_wasabi_cp_genome	AAGGGAAATATTGAATGAATTG
33_5_wasabi_cp_genome	ATCTCCAACCTAATCCAAAC
33_6_wasabi_cp_genome	TTGTTAGTTCATACAATGAATG
33_7_wasabi_cp_genome	AATTGCAACGATTCGATAAA
33_8_wasabi_cp_genome	AACTTATTGATTCGCACTAA
33_9_wasabi_cp_genome	ATTCCGTAATGACTGTACTCTG
33_10_wasabi_cp_genome	TACACAATCTCCAAGATCTTAC
33_11_wasabi_cp_genome	GACTTTGTTGAACTTACTCAATC
33_12_wasabi_cp_genome	CGATATACTGACTGAACTATGAC
33_13_wasabi_cp_genome	AAGGGTTAGAGACTACTCAATA
33_14_wasabi_cp_genome	CTAATAAATACCCTGCTAACAAC
33_15_wasabi_cp_genome	AATAGCATGTCGTATCAAGG
33_16_wasabi_cp_genome	GGAGTATATTTATGTACTTGCTT
33_17_wasabi_cp_genome	CTCAAGGATCCTTTCCTACA
33_18_wasabi_cp_genome	TTTGGTCAATCATTAATAATTG
33_19_wasabi_cp_genome	CTTCTGTAACTGGATAACCAG
33_20_wasabi_cp_genome	TTCGTTTTCATTCATTGTATG
34_1_wasabi_cp_genome	GATTAGTATAAATGTCGTTATGC
34_2_wasabi_cp_genome	CTCTTGTAAATGAAGGTATCTT
34_3_wasabi_cp_genome	ATTTGAGCAAACAATAGAGAA
34_4_wasabi_cp_genome	AGCCCCCATATATCAAACG
IR_1_wasabi_cp_genome	CCAAACTTTTCTGGTTTACC
IR_2_wasabi_cp_genome	CTTGAAACAATTTTGTCTTC
IR_3_wasabi_cp_genome	TCTGTAATGCATTGTATGTC
IR_4_wasabi_cp_genome	CTCGAATTTTTAGTATCCTAC
IR_5_wasabi_cp_genome	GTCAACTATTCAATCAAATG
IR_6_wasabi_cp_genome	GAAGAAAGAACAAGATGTTTC
IR_7_wasabi_cp_genome	CAACTTCGAATATGGAATTC
IR_8_wasabi_cp_genome	CATTAGTTCGTAGAGCTATTTAC
IR_9_wasabi_cp_genome	AGCCTATAGTGAGTTACAGAC
IR_10_wasabi_cp_genome	TATCAGACAATCACTTATTCAC
IR_11_wasabi_cp_genome	TATCGAAAGGCCTAATGAAT
IR_12_wasabi_cp_genome	ACAATATGCTACTACTGAAACA
IR_13_wasabi_cp_genome	CCATTCATTAGATAGAGAAGA
IR_14_wasabi_cp_genome	TATTAGTAAGAGGGATCTTGA
IR_15_wasabi_cp_genome	AGAAGAAGATGCCATTCATT
IR_16_wasabi_cp_genome	GATAGCATTTCTCTTCTGCA
IR_17_wasabi_cp_genome	GAAATATAACCAAGGTATATCTT
IR_18_wasabi_cp_genome	AATGGGAACTTGATGAGTTG
IR_19_wasabi_cp_genome	CTTGGACATATAAGAGTTCC
IR_20_wasabi_cp_genome	GAAGGGTACGAAATAAATTC

IR_21_wasabi_cp_genome	GAGTGAAATCTTTGTCTGCTA
IR_22_wasabi_cp_genome	CTACGGGTCCTGAACTTCTT
IR_23_wasabi_cp_genome	GTAAGGTGTTGGGTTAAGTC
IR_25_wasabi_cp_genome	CTTCCTATGGTAAGGAAGAG
IR_26_wasabi_cp_genome	CGTAGGAACTACGAGATCAC
IR_27_wasabi_cp_genome	GAATAGGTTAACCTTTTGAA
IR_28_wasabi_cp_genome	ACCGACTGATGTTGAAAAAT
IR_29_wasabi_cp_genome	TTAGCCGAAAGATGGTTATC
IR_30_wasabi_cp_genome	AGCCATCAGTGAGATACCAC
IR_31_wasabi_cp_genome	ATGGAGTGACAGAAGTTTTG
IR_32_wasabi_cp_genome	ACTTTCACATCTTCTTAACC
IR_33_wasabi_cp_genome	TCTTAATGGTCAAAGCGAAC
IR_34_wasabi_cp_genome	GTTCATATCGATCTATTATGC
IR_35_wasabi_cp_genome	GACAAAATTAATAAGGATGATGA
IR_36_wasabi_cp_genome	GAATAAAATGTGACCAATTAACC
IR_37_wasabi_cp_genome	TGGAATATAATGAAATAGAGC
IR_38_wasabi_cp_genome	GAAGGCCACTCCACTCTATT
IR_39_wasabi_cp_genome	CTCCATCCTTTGGATAGAAG
IR_40_wasabi_cp_genome	TCAGCCTGTTATCCCTAGAG
IR_41_wasabi_cp_genome	CATACATGGTCTTACGACTT
IR_42_wasabi_cp_genome	CTCGATCAGTGAGCTATTAC
IR_43_wasabi_cp_genome	AAGCTTACGGTTTCATGTTC
IR_44_wasabi_cp_genome	GTTCACGAGTTGGAGATAAG
IR_45_wasabi_cp_genome	CATCTTTGTCTCAGTAGAGTC
IR_46_wasabi_cp_genome	CTCGTAGTTCTTGGTCTGTG
IR_47_wasabi_cp_genome	GACTTCACTCCAGTCACTAG
IR_48_wasabi_cp_genome	CCTAGCTTTCGTCTCTCAGT
IR_49_wasabi_cp_genome	CTATTTCCAAAGGAACTGGA
IR_50_wasabi_cp_genome	GAATTCCTATTCGAAGAGTG
IR_51_wasabi_cp_genome	CCATACTCGATTTCATAGATAC
IR_52_wasabi_cp_genome	ATGGATCCATACATCTCGAT
IR_53_wasabi_cp_genome	GATCGGACTAATGACGTATAC
IR_54_wasabi_cp_genome	GAGACTCAAATGGTGGATATG
IR_55_wasabi_cp_genome	TATACATTCTCATTATGAGAAGG
IR_56_wasabi_cp_genome	TAAAGTACATTACATAGTCCGT
IR_57_wasabi_cp_genome	CTGTTACATTACATGAATCCTAT
IR_58_wasabi_cp_genome	ATCCTGAACCGATCTTACCT
IR_59_wasabi_cp_genome	GTTACTAGCATCAATATCGTCA
IR_60_wasabi_cp_genome	TTCTTATCAATGAATAGATCTC
IR_61_wasabi_cp_genome	TAGTATCCGCAATAGGTCTGA
IR_62_wasabi_cp_genome	TATTCAAGAATCGAAGTCGA
IR_63_wasabi_cp_genome	CATCGGACCATTTGTATCTA
IR_64_wasabi_cp_genome	CAAATCCCTTATGATACACC
IR_65_wasabi_cp_genome	CATTACTAATGGAATCGAAAGG
IR_66_wasabi_cp_genome	TAGATTCAGGCATGATACACTT
IR_67_wasabi_cp_genome	TCCAATAAGTCTATTGGAATTG
IR_68_wasabi_cp_genome	TATGGTAGAATCGTAACCATA
IR_69_wasabi_cp_genome	TCGTGTGGTACTTCTACCAT
IR_70_wasabi_cp_genome	AATAGAGAAGCTTAATACAAAGG
IR_71_wasabi_cp_genome	TATTTGTGAAATAACTTCGA
IR_72_wasabi_cp_genome	ATAGAACCCTGTTCAATAAG
IR_73_wasabi_cp_genome	CAGACCAAGAACTACGAGAT
IRa_12_F_wasabi_cp_genome	AGTTTCGAGAATATCCCCATTCATAGGT
IRa_12_R_wasabi_cp_genome	GTAAGAGGGGGAAGGGTTAAGGATTTAC

Supplementary Table S4. Primer sequences for the genotyping of wasabi major cultivars.

Primer Name	Primer sequence (5'–3')	*Tm*
mp_rpoC2_F_wasabi_cp_genome	TACTACAGAATCCCTTTGAA	54
mp_rpoC2_R_wasabi_cp_genome	CTTATTTAATCGATGAAATCA	54
mp_rbcL_300_F_wasabi_cp_genome	CATGGTATCCAAGTTGAAAG	57
mp_rbcL_300_R_wasabi_cp_genome	AGTCATGCATTACGATAGGA	57
indel_mazu_F_wasabi_cp_genome	TTCTAATTTTTTTAATTTCATTC	53
indel_mazu_R_wasabi_cp_genome	CATTTTATTTGCTAAATAACTCT	53
mp_18_mazu_F_wasabi_cp_genome	GCATGTCCACTGTTACTGTTA	58
18_45_wasabi_cp_genome	TCATTACGTGCGACTATCTC	58
Wasabi_cpSSR_J_ycf4_114F	ATTCTTATTGGTTGGAACTT	54
Wasabi_cpSSR_J_ycf10_1085R	TTCCACCAATTTGTAATCCAA	61
